# An integrated photonic device for on-chip magneto-optical memory reading

**DOI:** 10.1515/nanoph-2022-0165

**Published:** 2022-06-15

**Authors:** Figen Ece Demirer, Yngwie Baron, Sander Reniers, Dzmitry Pustakhod, Reinoud Lavrijsen, Jos van der Tol, Bert Koopmans

**Affiliations:** Department of Applied Physics, Eindhoven University of Technology, Eindhoven, The Netherlands; Department of Electrical Engineering, Eindhoven University of Technology, Eindhoven, The Netherlands

**Keywords:** ferromagnetic thin-films, integrated photonics, MOKE, non-volatile memory, photonic memory, polarization conversion

## Abstract

This study presents the design, fabrication and experimental demonstration of a magneto-photonic device that delivers non-volatile photonic memory functionality. The aim is to overcome the energy and speed bottleneck of back-and-forth signal conversion between the electronic and optical domains when retrieving information from non-volatile memory. The device combines integrated photonic components based on the InP membrane on silicon (IMOS) platform and a non-volatile, built-in memory element (ferromagnetic thin-film multilayers) realized as a top-cladding on the photonic waveguides (a post-processing step). We present a design where the phase of the guided light is engineered via two mechanisms: the polar magneto-optical Kerr effect (MOKE) and the propagation in an asymmetrical cross-section (triangular) waveguide. Thanks to its design, the device yields different mode-specific transmissions depending on the memory state it encodes. We demonstrate the recording of the magnetic hysteresis using the transmitted optical signal, providing direct proof for all optical magnetic memory reading using an integrated photonic chip. Using mathematical model and optical simulations, we support the experimental observations and quantitatively reproduce the Kerr signal amplitudes on-chip. A 1% transmitted power contrast from devices is promising indicating that in a shot noise limited scenario the theoretical bandwidth of memory read-out exceeds 50 Gbits/s.

## 1 Introduction

Our modern-day civilization consumes and generates data at an exponentially increasing rate, demanding an ever-growing computational power and bandwidth. This fuels the technological advancements towards faster, cheaper and more energy efficient operations. As previous research shows [1–4], incorporating integrated photonics with electronic circuity offers a drastic performance increase in data-com and tele-com. Yet, new bottlenecks form at points of signal conversion between the electronic and optical domains. For certain operations, the electronic and optical circuitries have varying performance levels, which implies that using conversions between the two domains (electronic and optical) is a requirement for improving the overall system performance. To exemplify, certain applications leverage the faster signal-transfer in the optical domain by using photonic interconnects, while the memory-related tasks such as storage and read/write operations still take place in the electronic domain [5]. Especially in data centers and neural network training operations, for which frequent non-volatile memory retrieval is required [6], we anticipate a huge potential when using all-optical operations, cutting down on intermediate electronics steps, saving time and energy. To unlock this potential, integrated photonic components that deliver optical memory functionality through various different mechanisms have been suggested [7–9]. However, photonic memory alternatives are currently out-performed by their electronic counterparts. Considering the high storage density of conventional magnetic non-volatile memories and their well-established wafer-scale production capabilities, we believe that it is a strategical next-step to invest in the field of integrated magneto-photonics. Since light and conventional magnetic memory materials interact magneto-optically [10] and yield a distinct signal depending on the memory state, such materials can be candidates for an optical memory in integrated photonic circuits. Regarding the interaction of light with the magnetic memory materials, there are ongoing efforts on establishing all-optical switching of spintronic memory bits that are based on films with perpendicular magnetic anisotropy (PMA) [11–15]. The reported experimental evidence is mostly based on free-space optics, except one study that proposed all-optical memory writing of non-volatile magnetic random-access memory (MRAM) in an integrated photonic device setting [16]. Despite these developments, to our knowledge, there is no study focusing on the magneto-optical reading of the magnetic memory using a hybrid integrated photonic device that implements magnetism into the picture. Considering these factors, we aimed to demonstrate a proof-of-principle device that combines the fields of spintronics and photonics to deliver an on-chip magneto-optical memory reading functionality.

Our proposed magneto-photonic device carries a built-in, non-volatile, nano-scale magnetic memory bit that is applied to its waveguide as a top-cladding. The cladding is a ferromagnetic, multilayered ultrathin-film (total thickness of 12  nm) containing Co/Pt layers with PMA [17]. Our choice for this system is motivated by the aforementioned combination of relatively large magneto-optical effects, the availability of efficient all-optical switching scenarios and PMA magnetic thin film systems being the preferred system in today′s spintronics. As to the latter, materials with PMA dominate the non-volatile memory applications, such as spin-transfer torque (STT) MRAM, thanks to their increased memory density and thermal stability [18]. Although state-of-the-art PMA structures have been shown to interact with optical pulses to store information in magnetic memory [14] and are known for their relatively large magneto-optical efficiencies, they have not been explored for optical memory applications yet. The working principle of our magneto-photonic device for delivering the magneto-optical memory reading functionality is the polar magneto-optical Kerr effect (polar MOKE) which takes place when the guided light interacts with the PMA top-cladding [19]. While MOKE by metallic magnetic claddings in longitudinal and transverse configurations has been well investigated [20, 21], the potential for this polar configuration has been overlooked to date.

The fabricated device has its photonic components based on the InP membrane on silicon (IMOS) platform [22], where the relatively weak confinement of light within the InP membrane ensures a stronger interaction between the light and the magnetic cladding. In this setting, MOKE causes a change in the polarization state of the guided light, which changes sign when the magnetization direction of the memory component is reversed [23]. In a photonic waveguide, this gives rise to a partial mode conversion between the TE and TM modes, where the phase of the emergent mode carries the information about the magnetization direction of the cladding, thus its memory state. More specifically, in the case of a TE mode partially converted into TM mode, the relative phase of the modes will change by *π* (180°) upon reversal of the magnetization direction. Considering that the information is stored in the phase of the guided light, we propose a novel device design where the change in polarization state is converted to an intensity contrast. Additionally, such a device can be used to quantitatively determine the intrinsically small MOKE [10] when combined with mathematical models.

This paper is structured in the following way. In Section 2 the methodology is provided, describing the design, mathematical modelling, fabrication and optical characterization of the magneto-photonic devices. Next, in Section 3, the experimental evidence demonstrating the proof-of-principle for the magneto-optical memory reading is demonstrated and the on-chip MOKE amplitude is quantitatively determined. Additionally, the optical simulation results are presented and compared with the experimental evidence. Finally, in Section 4 the conclusions are drawn.

## 2 Methodology

### 2.1 Design and modelling

A magnetic cladding placed on a waveguide gives rise to the MOKE that is observed as an intrinsically small mode conversion between the eigenmodes (TE and TM) of the waveguide. Depending on the magnetization direction of the cladding (up- or downwards magnetized), the mode conversion occurs with a phase difference of *π* albeit with the same amplitude. In other words, the phase of the emergent mode encodes the information regarding the magnetization direction of the cladding, thus its memory state. However, since the relative phase is difficult to directly extract, we aimed at a design that yields two different transmitted light output intensities depending on the magnetization direction of its cladding.

**Figure 1:**
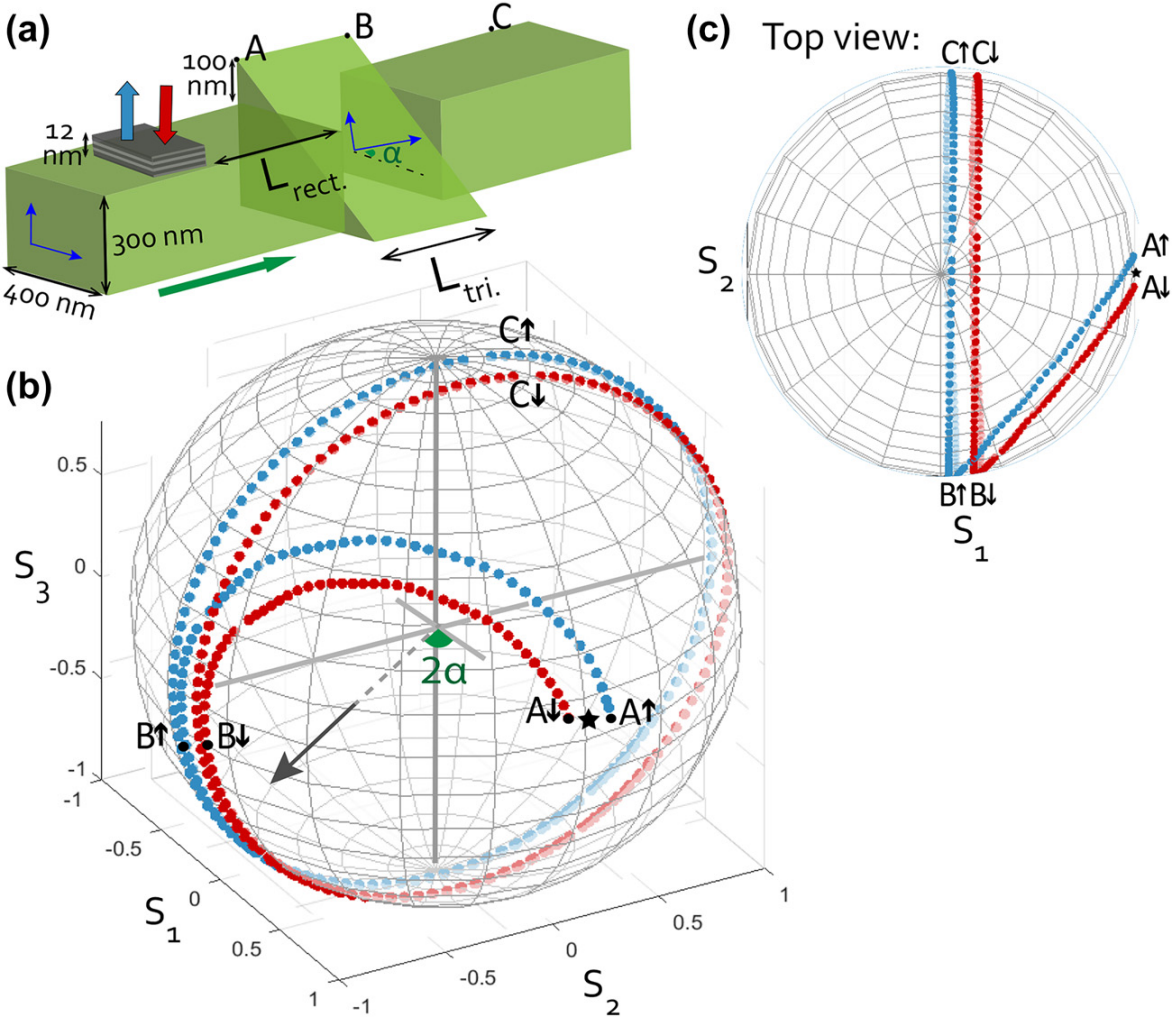
(a) The core-module of the magneto-photonic device, showing the magnetic memory component (multi-layered top-cladding) and the polarization rotator component (triangular waveguide). The two memory states (magnetization directions) are indicated with the red and blue arrows. The birefringence in the triangular section is indicated by the tilt of its eigenmodes axes by an angle *α*. (b, c) Poincaré sphere [24] representations showing the polarization state evolution throughout the device. Plots are colour-coded (blue and red), indicating the magnetization direction of the cladding the light has interacted with (up and down, respectively.). Note that the chosen Kerr amplitude in the plots is for visualization purposes thus does not reflect the experimental results quantitatively.

We present the design in its two formats: a core-module and a stand-alone device. The former solely contains the memory component and a polarization rotator element. Thus, it can provide the magneto-optical memory reading functionality, only when implemented in an integrated photonic environment. The stand-alone device includes the core-module and mode-selective grating couplers to couple light in from an external light source, and couple it out to a detector. It is tailored for proof-of-principle measurements. Figure 1(a) depicts the core-module, while (b) and (c) demonstrate its functioning principles using the Poincaré sphere representation. Depicted in (a) as the top-cladding, the information is stored in a multi-layered, ferromagnetic, thin-film memory material that has Co/Pt bilayers on a Ta seeding layer. The triangular waveguide section acts as a partial polarization converter [25] that is referred to in this work as a polarization rotator.

Let us describe the functioning principles of the core-module using the representations in Figure 1b and c. Eigenmodes in different sections are referred to as the TE and TM modes of those sections. On the sphere, each point corresponds to a unique polarization state, indicated with the Cartesian co-ordinate system where *S* indicates the Stokes parameters [26]. The *S*_1_-axis component relates to how much power is in the TE (*S*_1_ = 1) or TM (*S*_1_ = −1) mode. The *S*_2_ and *S*_3_-axes components describe the phase relation between the two modes. If the guided light is a mixture of the two modes, a mode beating occurs during propagation due to the difference of the effective refractive indices (or propagation constants) of the two modes. One period of beating length in a waveguide with cross-section cs is expressed as

(1) $$L_{beat.,\;cs}=\frac{\lambda }{\;|\Delta n_{e\mathrm{ff}.,\;cs}|\;},$$

where *λ* is the vacuum wavelength of the light and Δ*n*_eff.,cs_ is the difference in the effective refractive indices between the eigenmodes of that cross-section. As Figure 1b and c show, the TE-mode input (*S*_1_ = 1, *S*_2_ = *S*_3_ =0), indicated with a star, propagates undisturbed (no mode-beating) until the region with the magnetic cladding is reached. Here, the MOKE takes place, yielding a small mode conversion described by the Kerr rotation (*θ*) and ellipticity (*ɛ*). Note that the attained complex MOKE signal (Φ) is slightly different for the TE and TM modes due to the difference in interaction between the cladding and the TE and TM modes. The subscript TX indicates the mode is TE or TM.

(2) $$\Phi_{\text{TX}}=m\;\left(\theta_{\text{TX}}+i\;\varepsilon_{\text{TX}}\right).$$

In our devices TE mode is used. The up- and down-ward magnetized claddings are indicated by *m* = ±1. The equation reflects the statement that the mode conversion is equal in amplitude but with a phase difference of *π* for the two memory states. Upon attaining a mixed-mode state due to MOKE, a mode-beating occurs while propagating in the rectangular waveguide section through the length *L*_rect*.*_. The beating is omitted in the figure for clarity. The triangular waveguide section has its unique eigenmodes that are tilted by an angle *α* with respect to the ones in the rectangular section. When the guided light enters the triangular waveguide section at point A (see Figure 1b and c), the modes get projected onto the tilted eigenmodes of this section. The projected states are marked by the points A ↑ and A ↓. The arrows indicate the magnetization orientation of the cladding thus the memory state and is represented by *m* = ±1 elsewhere. As the mixed-mode travels in this section, the mode beating is observed as circling around the axis which makes an angle of 2*α* with respect to the *S*_1_-axis (see arch $\overset{⌢}{AB}$ ). After propagating over a distance *L*_tri._, which is chosen to be equal to the half beat length of the triangular section, the states B ↑ and B ↓ are obtained. Coupling back to the rectangular waveguide, the phase evolution continues as shown by the arch $\overset{⌢}{BC}$ . Comparing the initial polarization state (⋆) with the final states (C ↑ and C ↓) -in terms of TE and TM mode contributions highlights the working principle of the presented design. Figure 1b and c geometrically illustrate that, prior to propagation in the triangular section; the states that correspond to the magneto-optic interaction with the up- and down-magnetized claddings have the same relative power in the TE and TM-modes of the rectangular section (equal *S*_1_ components of A ↑ and A ↓). After the triangular section, a difference in the relative mode-power is created, i.e., the *S*_1_ components are different for C ↑ and C ↓. This difference provides the magneto-optical memory reading functionality. The figure provides an intuitive picture that by engineering the rectangular and the triangular section lengths, the mode intensity contrast can be maximized.

**Figure 2:**
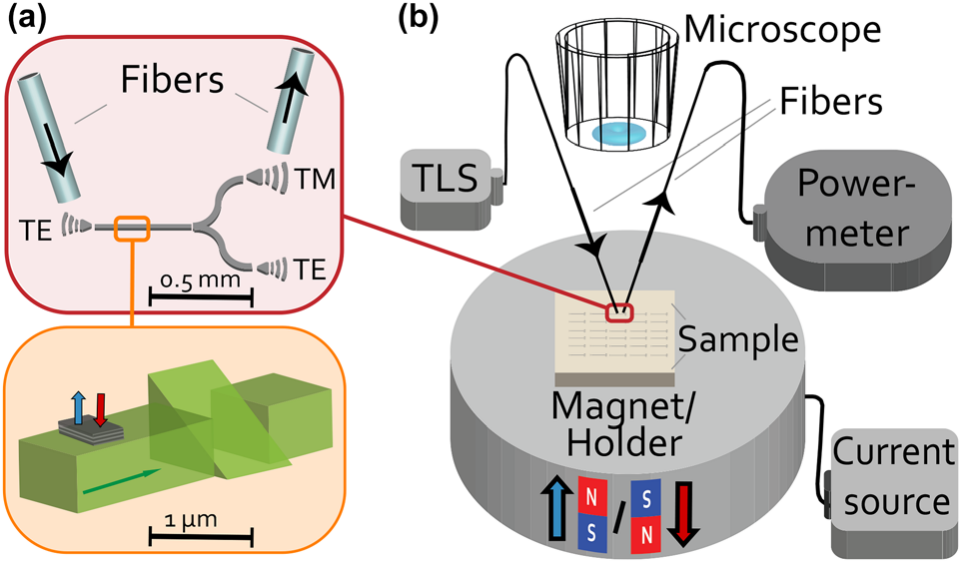
(a) A schematic of the proof-of-principle type, stand-alone magneto-photonic device. Top: Overall device with mode-selective (TE and TM) grating couplers, waveguides and splitters are shown. Bottom: A zoom-in view of the core-module, showing the memory component (top-cladding) and the polarization rotator (triangular waveguide). (b) The optical transmission measurement set-up. A tunable laser source (TLS) sends the light (*λ* = 1550 nm) through the input fiber. After transmission through the device, the output fiber collects the light and directs it to the optical power meter. The sample holder is an electro-magnet that can apply up to ±300 mT perpendicular magnetic field, setting the magnetization direction of the memory component on-demand. The fibers are not polarization maintaining and are cleaved to enable an easier coupling.

The second format of the design, the stand-alone device form, is designed for the proof-of-principle measurements. A mathematical model based on the Jones formalism [27] assisted with the design and the analysis of the experimental observations. Depicted in Figure 2a, the device has a TE mode-selective in-coupler and TE and TM mode-selective out-couplers. As seen in Figure 2b, the optical fibers connect the couplers to an external laser and an optical power meter. In the mathematical model that describes this device, the MOKE matrix **M** is defined as

(3) $$M=\left(\begin{array}{cc} 1 & -\Phi_{\text{TE}}\\ \Phi_{\text{TM}} & 1 \end{array}\right),$$

where Φ_TX_ is the complex Kerr signal (see Eq. (2)) experienced by the TE or TM-mode inputs. We note that the defined MOKE matrix is an over-simplification, only valid when the input is a pure mode and the Kerr signal is small in amplitude (which is always the case). The phase evolution of the light due to propagation in a waveguide with a certain cross-section is indicated by the matrix **P**_cs_.

(4) $$P_{\text{cs}}=\left(\begin{array}{cc} e^{\;i\frac{2\pi }{\lambda }n_{\text{a, cs}}L_{\text{cs}}} & 0\\ 0 & e^{\;i\frac{2\pi }{\lambda }n_{\text{b, cs}}L_{\text{cs}}} \end{array}\right),$$

where *n*_a,cs_ and *n*_b,cs_ are the effective refractive indices of the modes that are defined as the horizontally (in-plane) oriented electric and magnetic fields in that particular cross-section. The waveguide length with the corresponding cross-section is indicated with *L*_cs_. To describe the propagation in the triangular section, the axis of interaction is tilted back and forth via the rotation matrix **R**. This tilt is required in order to align the eigenmodes of this section with the axis of interaction. It is formulated as

(5) $$R=\left(\begin{array}{cc} \cos\alpha & \sin\alpha \\ -\sin\alpha & \cos\alpha \end{array}\right),$$

where *α* is the tilt angle. Correspondingly, the resulting mode-specific output electric field $E_{{\text{TX}}_{o}}$ is formulated as

(6) $$\left(\begin{array}{c} E_{{\text{TE}}_{o}}\\ E_{{\text{TM}}_{o}} \end{array}\right)=R\left(-\alpha \right)\cdot P_{\text{tri.}}\cdot R\left(\alpha \right)\cdot P_{\text{rect.}}\cdot M\cdot \left(\begin{array}{c} E_{{\text{TE}}_{i}}\\ E_{{\text{TM}}_{i}} \end{array}\right),$$

where $E_{{\text{TX}}_{i}}$ is the input E-field. For the sake of simplicity, in all matrices the optical losses are neglected, but including them would not change the generic principle. Since the mode-specific light intensity *I*_TX_ is proportional to the output light’s field amplitude square $|E_{{\text{TX}}_{o}}{|}^{2}$ , we calculate the mode-specific intensity contrast (Δ*I*_TX_) in units of dB as

(7) $$\Delta I_{TX}=10\;{log}_{10}\left(\frac{|\;E_{{TX}_{o},\;\;m=1}\;{|}^{2}}{|E_{{TX}_{o},\;m=-1}{|}^{2}}\right).$$

Equation (7) accounts for the difference in transmission when the memory state of the device is changed. Despite the optical losses are ignored, a quantitative analysis using Eq. (7) is possible since it is in the ratio form.

**Figure 3:**
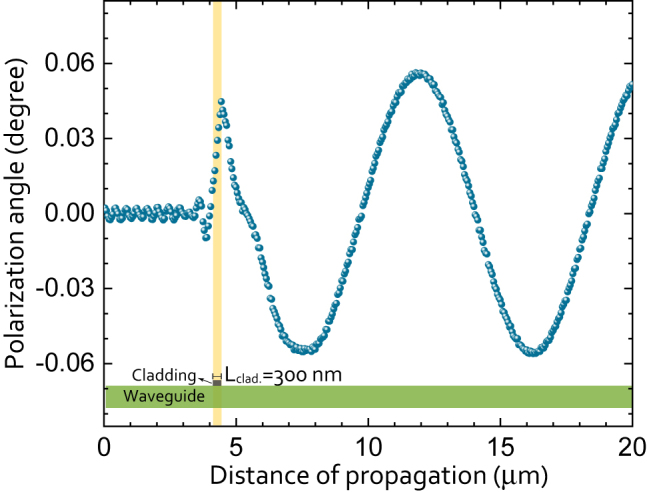
Optical simulation (FDTD), showing the polarization evolution of the initially purely TE-polarized input light, as it propagates in the rectangular waveguide section cladded with the memory material. The polarization angle refers to the rotation caused by the MOKE. The region with the magnetic cladding (memory) is shown with the vertical highlighted region. The stack order and the length of the cladding are Ta4/Pt2/Co1/Pt2/Co1/Pt2 (numbers in nm) and 300 nm, respectively. A conformal meshing of 2 nm is used.

Lastly, we present an optical simulation based on finite difference time domain (FDTD) method [28–30] that demonstrates the polarization angle evolution as a function of the propagation distance. The simulation uses material properties, i.e., the refractive indices and the magneto-optical constants that were previously obtained experimentally for this specific material set [31]. Figure 3 shows an initially fully TE-polarized input mode that interacts with a 300  nm-long magnetized top-cladding. According to the simulations, the amplitude of the complex MOKE (|Φ_TE_|) is 0.06° ± 0.02°. As will be discussed in Section 3, this is in good agreement with the experimental findings. In Figure 3, the highlighted region indicates the length covered by the cladding. In this region, the Kerr rotation increases cumulatively. The simulation accounts for both forward and backward propagating waves. The Kerr rotation observed within the first μm of propagation after the cladding suggests that there are non-confined (higher order) modes, possibly excited by the cladding, which also show a finite MOKE. Higher order modes reflected (backwards propagating) from the cladding show the same behaviour. After these higher-order modes decay, a purely oscillatory polarization (due to MOKE on the primary mode) state is captured by the simulation. The beating periodicity of 8.1 μm indicates an effective index difference (|Δ*n*_eff. TE_ − Δ*n*_eff. TM_|) of 0.19 (see Eq. (1)). This value is close to what can be calculated for the specific rectangular InP waveguide of choice, using literature values for the refractive index. In the region prior to the cladding, oscillations in Kerr rotation with higher frequency and lower amplitude are observed. They are attributed to an interference including the reflections from the cladding, which also experience a MOKE.

### 2.2 Fabrication

The magneto-photonic devices are based on the IMOS platform [22]. The devices have varying lengths of rectangular and triangular waveguide sections (*L*_rect*.*_ and *L*_tri*.*_, respectively), and two magnetic cladding lengths of 300 and 600  nm are used (albeit with an equal width of 400  nm, which is the same as the waveguide width).

Briefly describing the fabrication process, the photonic structures are defined with multiple e-beam lithography (EBL) steps, followed by wet- and dry-etching steps of the InP membrane. The etching steps are based on acid solution etching and reactive ion etching via inductively coupled plasma, respectively. The sloped side-walls of the triangular waveguides are obtained with wet-etching, where the $\bar{1}\bar{1}2$ crystalline planes naturally terminate the etch [32]. The vertical side-walls are realized with dry-etching. The thickness of the triangular and rectangular sections are 460 and 300 nm, respectively. For details on the fabrication process, we refer the reader to a previous work by two of our co-authors [33]. The memory components are applied on top of the waveguides as claddings. To define the area of the memory bit, an additional overlay exposure EBL step is used. After ensuring a clean opening on the InP membrane, the multi-layers are deposited using magnetron sputtering, further explained in [31]. The stack order of the multi-layer is Ta4/Pt2/Co1/Pt2/Co1/Pt2, numbers indicating the layer thickness in nm. After the metal deposition, a lift-off step is performed to remove the excess metal film from the devices.

Characterization of the photonic components, as described in Appendix A, revealed that the effective refractive index differences in rectangular (Δ*n*_eff.,rect._) and triangular waveguide sections (Δ*n*_eff.,tri._) are 0.25 and 0.34, respectively; while the angular tilt of the eigenmodes in the triangular section (see *α* in Figure 1a) is 21°.

### 2.3 Measurement set-up

Optical transmissions from the stand-alone devices are measured in the set-up depicted in Figure 2b. Since the magneto-photonic devices are designed to yield different mode-specific outputs for opposing memory states, the set-up is designed to probe the optical transmission while switching the magnetization direction of the memory component. Therefore, the set-up included a fiber coupled laser source (*λ* = 1550 nm), an optical power meter and an electromagnet sample holder connected to a current source allowing to apply perpendicular magnetic fields up to 300 mT in up- and downward directions. The applied field is used to set the magnetization direction of the cladding, thus its memory state.

Two different measurement procedures are followed. The first one is aimed at hysteresis measurements, where a strong field is applied in a certain direction at the beginning to set the magnetization direction of the cladding. Then, the magnetic field is incrementally swept towards both directions, and the optical transmission is probed. The second method, the contrast measurement, is focused on probing the difference in the light transmission between the remnant up- and down-states. In this method, the magnetization direction of the cladding (the memory bit) is periodically switched between the up- and downwards magnetized states by applying fields with equal amplitude but opposite sign. The transmitted light intensity is recorded after setting of the magnetization direction. Note that in both procedures, the transmission is measured after removing the externally applied magnetic field. Despite this, the magnetization of the thin-film is preserved due to its non-volatility.

## 3 Results and discussions

As described earlier, the magneto-optical memory reading functionality is achieved by designing devices that yield different mode-specific transmissions depending on their cladding’s memory state. In this section, we present the measurements obtained from such devices. The model (presented in Section 2.1) predicted the dependence of the mode-specific transmission contrast on certain device parameters. We provide evidence that this is indeed the case and the MOKE for light confined in waveguides can be quantitatively determined using this model. Lastly, we compare the reported experimental findings with the previously presented optical simulation results (Section 2.1).

**Figure 4:**
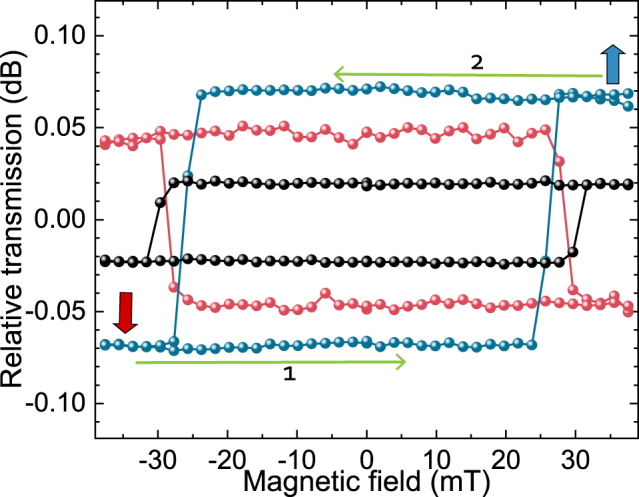
Hysteresis behaviour observed in the mode-specific transmission as a function of the applied magnetic field when fully TE-mode input light is used. Plots show different magneto-photonic devices (with varying lengths of *L*_rect._ and *L*_tri._), albeit with the same magnetic cladding length of 600 nm. Prior to the measurements, the memory state of the cladding is set by applying −40 mT field. Then, the magnetic field is swept in the direction and order indicated by the horizontal arrows. The jumps in the relative transmissions (at approximately ±30 mT), correspond to the switching of the magnetization direction of the claddings, thus changing of the memory states. The combinations of (*L*_rect._, *L*_tri._) length parameters in μm are (7.77, 4.3), (7.77, 1.0) and (5.07, 2.2); for blue, red and black data-sets, respectively.

Figure 4 shows the relative TE-mode transmission (in units of dB) from three different devices as a function of the applied magnetic field. The hysteresis behaviour is clearly observed. As mentioned in Section 2.3, the built-in memory components (top cladding) of the devices are set to downward magnetized states by applying a magnetic field of −40 mT prior to the measurements. As indicated with horizontal arrows in the figure, the applied field is swept in both directions, creating the depicted loops. The changes in the mode-specific transmission at the coercive fields of approximately ±30 mT, indicate that the magnetization direction of the built-in memory element is changed. The abruptness of the changes indicates that the claddings have PMA, thus the preferential magnetization orientation lays out-of-plane. This was confirmed using polar MOKE measurements ex-situ, in a free space optics set-up i.e., with light incident from above. To our knowledge, this is the first time hysteresis behaviour is observed on-chip for such a sub-micon magnetic memory element. These experimental findings prove that the magneto-optical memory reading in integrated photonic circuits is possible by using such designs with a reasonable signal-to-noise ratio (SNR). Additionally, Figure 4 demonstrates that the amplitude and the sign of the obtained contrast depend on the length parameters (*L*_rect._ and *L*_tri._) of the device. This observation is in accordance with the predictions of the model (as can be derived from Eqn. (6)).

**Figure 5:**
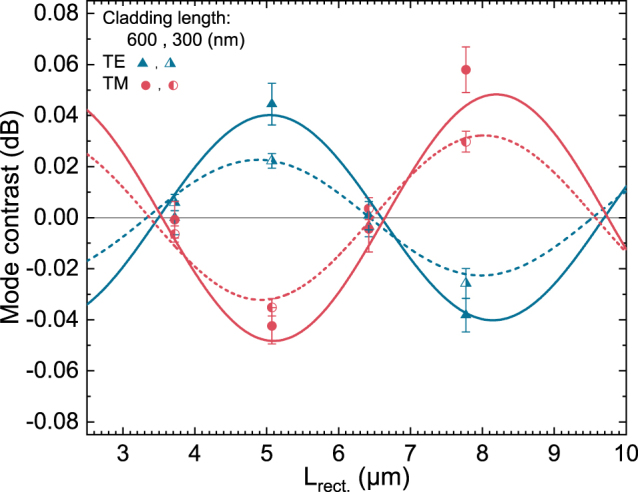
Mode-specific transmission contrasts (Δ*I*_TX_) obtained from devices with different rectangular waveguide section lengths *L*_rect._. The mode contrast (Eqn. (7)) refers to the difference in transmission when the memory state is changed (when the magnetization direction of the cladding is switched). Devices with the magnetic cladding lengths of 300 nm (half-filled points) and 600 nm (filled points) are reported. All devices have a triangular section length *L*_tri._ of 1.8 μm. While the data-points are the experimentally obtained values, the lines (dashed or solid) indicate the predictions for a certain MOKE signal (Eq. (7)). *L*_rect._ refers to the distance between the magnetic cladding and the triangular section (see Figure 1a).

**Figure 6:**
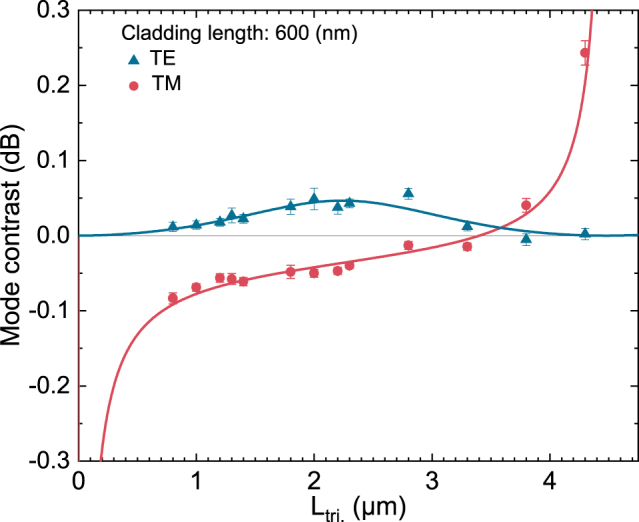
Mode-specific transmission contrasts (Δ*I*_TX_) obtained from devices with different triangular waveguide section lengths (*L*_tri._). The mode contrast refers to the difference in transmission when the memory state is changed (when the magnetization direction of the cladding is switched). All devices have a rectangular waveguide section length *L*_rect._ of 5.07 μm, and a cladding length *L*_clad._ of 600 nm. The data-points indicate the experimentally obtained values while the solid lines are the predictions for the fitted MOKE signal (Eq. (7)).

Further evidence to the dependence of the mode-specific contrast on certain device parameters is provided by Figures 5 and 6. Here the contrast refers to the difference in the mode-specific transmission (Δ*I*_TX_) in units of dB; more specifically, the difference in output intensity between the up- and down-wards cladding magnetization directions. In the figures, the mode-specific contrasts (Eq. (7)) are plotted as a function of the rectangular and triangular waveguide section lengths (*L*_rect._ and *L*_tri._), respectively. The figures depict the experimentally obtained values and the fits to the model predictions with the separate data-points and the dashed/continuous lines, respectively. Figure 6 shows an asymptotic behaviour for the TM-specific mode contrast as the *L*_tri._ approaches to 0 and 4.5 μm. This is a result of the diminishing overall TM-mode transmission from the devices. When the length of the triangular waveguide (polarization rotator) is *L*_tri._ = 0 μm or *L*_tri._ = *L*_beat._, the TM-mode transmission from the devices vanishes and thus the relative contrast diverges.

**Figure 7:**
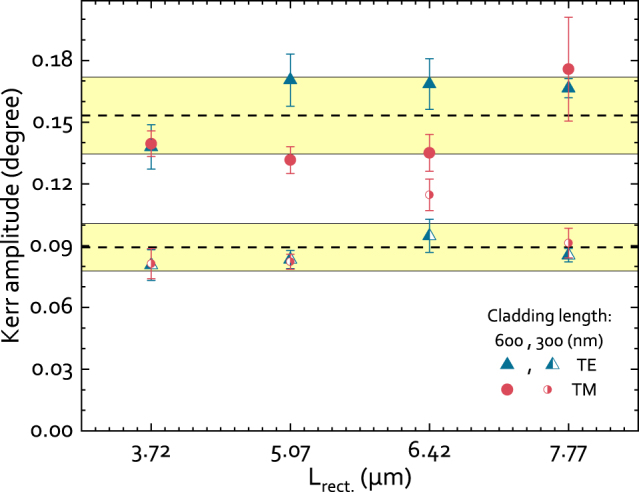
Kerr amplitudes (Φ_TX_) determined from devices with varying propagation lengths in the rectangular waveguide section (*L*_rect._). The results from devices with two different cladding lengths (*L*_clad._ = 300 and 600 nm) are shown. Each data-point is the result of a fitting procedure where 4 devices with varying *L*_tri_ are used. The error bars indicate the accuracy of the fits. The dashed horizontal lines show the average Kerr amplitude, while the widths of the highlighted regions indicate the standard deviation of the reported data-points.

Using the experimental evidence of mode contrasts from devices with varying length parameters, we quantitatively determined the magneto-optical activity in terms of the MOKE amplitude, as it is often the reported figure-of-merit [34]. The amplitude of the complex value is calculated as $\sqrt{{\theta_{TX}}^{2}+{\varepsilon_{TX}}^{2}}$ . The resulting amplitudes for a large number of devices with different parameters are summarized in Figure 7. The standard deviation amongst the obtained values is indicated by the width of the highlighted region. Results show that the Kerr amplitude |Φ_TX_| doubles (within the error margin) upon doubling of the cladding length *L*_clad._ (300–600  nm). The linear dependence of the mode contrast Δ*I*_TX_ on *L*_clad._ is in accordance with the predictions by FDTD simulations (not shown here). The simulations predict that the linear relation holds for *L*_clad._ up to the half a beat length in the rectangular waveguide section *L*_beat.,rect._. The experimentally obtained MOKE amplitude |Φ_TE_| of 0.09° ± 0.02° for *L*_clad._ = 300  nm closely matches with the simulated value of 0.08° ± 0.02° based on previously reported [31] values of (magneto-) optical parameters (see Figure 3). The optical loss was found to scale as well linearly with *L*_clad._. In the current configuration, the experimentally obtained loss per 100  nm is 0.55 dB for the TE mode.

Regarding the noise in the experimental results, the major source of instability is found to be the stochastic movement of the optical fibers over the grating couplers (see Figure 2a). In a future application where the core-module is combined with a built-in laser and detector, the noise from the moving parts would be eliminated, allowing the SNR to improve many orders of magnitude. In order to place such a prospect device on the map with respect to the current technologies, and demonstrate its promises in terms of bandwidth of data read-out, we assumed an ideally stable configuration and perfect (opto-)electronical components, where the device is limited by shot noise. Accordingly, to resolve the 1% contrast that the current device delivers, we calculated the theoretical limit of the maximum data rate to be better than 50 Gbits/s (see Appendix 6). Assuming the memory is implemented in the form of a racetrack memory [15, 35], for which rapidly increasing domain wall velocities have been reported [36, 37], the prospect device competes well with the optical [38] and electrical [39] counterparts that currently offer tens of Gbits/s and few Gbits/s [5], respectively.

## 4 Conclusions

For the first time in literature, we demonstrated magneto-optical reading of a non-volatile magnetic memory in an integrated photonic device setting. This was achieved via the designed hybrid magneto-photonic devices implemented in IMOS. Devices were engineered such that the guided light yields a difference in the mode-specific optical transmission upon changing of the memory state up to 1%. With this, a typical hysteresis behaviour observed in magnetic materials is demonstrated in an on-chip setting. It is highlighted that our photonic memory material of choice having out-of-plane magnetic orientation offers the advantages of enhanced MOKE in this polar configuration and higher memory storage densities. Additionally, the multi-layered ferromagnetic thin-films are well-established for spintronic memory applications and compatible with the PIC-technology.

We developed a mathematical model that describes the transmitted mode intensity in terms of device parameters, and which assisted the design process and helped to interpret the experimental observations. Thanks to the model, the quantitative determination of the MOKE in integrated photonic devices was possible, which enabled a quantitative comparison with the optical simulations. The simulations that utilize the magneto-optical constants we previously determined [31], successfully reproduced the experimental observations and provided insights into the MOKE for light confined in waveguides. This is significant since it validates the determined magneto-optic activity in ultrathin-films and builds confidence on the results from simulations.

As a technological outlook, a theoretical limit of memory read-out bandwidth of tens of Gbits/s is predicted based on the current device designs. We discussed that establishing a photonic memory that can compete with the electronic counterparts has the potential of drastically improving the data-com and tele-com operations, cutting down on the requirements of back-and-forth electronic to optical signal conversions. Such a device is envisioned to directly generate amplitude modulated photonic bit patterns out of the magnetic memory, avoiding any energy and time consuming high-frequency electronic operations.

## Figures and Tables

**Figure 8:**
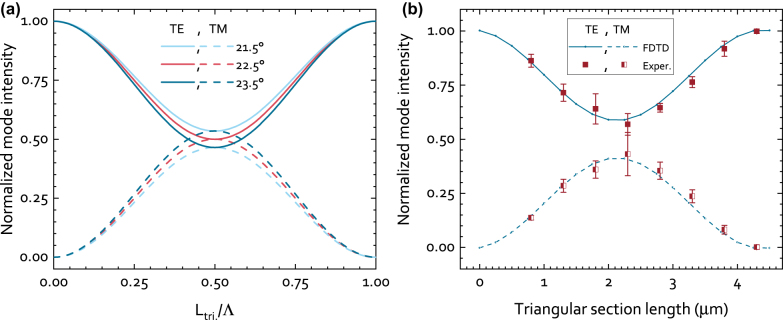
(a) Modelled mode conversion as a function of triangular cross-section waveguide length showing the case for different values of *α*. Note that the beating length $L_{{beat.}_{\text{cs}}}$ is indicated by Λ. (b) FDTD simulation and experimental evidence showing mode conversion as a function of triangular cross-sectioned waveguide length. Error bars are defined by measuring 3 sets of identical devices. Both graphs assume TE mode input.

## Appendix A: Characterization of the photonic components

Characterization of the light transmission of the photonic devices has been performed during the fabrication process, more specifically after the fabrication of the photonic structure and before the placement of the magnetic claddings.

The mathematical model predicts that the triangular waveguide section provides a partial mode conversion where the electric fields of TE or TM modes are expressed as

(8)
$$\left(\begin{array}{c} E_{{\text{TE}}_{o}}\\ E_{{\text{TM}}_{o}} \end{array}\right)=R\left(-\alpha \right)\cdot P_{\text{tri.}}\cdot R\left(\alpha \right)\cdot \left(\begin{array}{c} E_{{\text{TE}}_{i}}\\ E_{{\text{TM}}_{i}} \end{array}\right).$$

The mode intensities are

(9)
$$\begin{array}{l} \begin{array}{cc} |E_{{\text{TM}}_{o}}{|}^{2} & =sin{\left(2\alpha \right)}^{2}\sin{\left(\frac{\pi }{\lambda }\;\Delta n_{\text{tri.}}\;L_{\text{tri.}}\right)}^{2},\\ |E_{{\text{TE}}_{o}}{|}^{2} & =1-|E_{{\text{TM}}_{o}}{|}^{2}, \end{array} \end{array}$$

where Δ*n*_tri._ is the difference between the TE and TM modes effective refractive indices. The mode-specific normalized light intensities from these devices without the magnetic claddings are shown in Figure 8a and b. Figure 8(a) depicts the mathematical model predictions while (b) shows the experimental evidence combined with the FDTD simulations. By using Eq. (9) to interpret the data in Figure 8b, we found Δ*n*_tri._ = 0.34 and *α* = 21.02°. Note that the experimental observation of the mode beating behaviour in the rectangular section is used for the determination of Δ*n*_rect._ = 0.25, which is slightly different than the (FDTD) simulated value (0.19). This is expected to be due to the side-wall roughness that is ignored during the simulations.

## Appendix B: Theoretical bandwidth limit of the memory read-out

For a magneto-photonic device using the design presented in this work as a core-module, we calculated the theoretical memory reading bandwidth. We assumed that the device is limited by the shot noise to calculate the theoretical SNR. Using such estimation we determined if the proposed memory read-out device is competitive with alternative optical and electrical memory devices. Using the experimentally obtained Kerr rotation *θ*_TX_ and phase *ɛ*_TX_ for *L*_clad._ = 600 nm, we calculated the E-field amplitudes at the output of the device. The SNR is in this case given by

(10)
$$SNR=\sqrt{\frac{P_{in}t}{E_{ph}}}\frac{\left|\;|E_{{TX}_{\text{o}},\;\;m=1}\;{|}^{2}-|\;E_{{TX}_{o},\;\;m=-1}\;{|}^{2}\right|}{\sqrt{\frac{1}{2}\left(|\;E_{{TX}_{o},\;\;m=1}\;{|}^{2}+|\;E_{{TX}_{\text{o}},\;\;m=-1}\;{|}^{2}\right)}},$$

where *P*_in_ is the input power, *E*_ph_ is the photon energy at a wavelength of 1550  nm and *t* is the duration in which the laser is on. Assuming an SNR of 6 would suffice to resolve the signal from the noise; a laser input power of 5 mW revealed the duration *t* that is required for the measurement to succeed. The time restriction indicated a read-out speed of about 60 Gbits/s. We emphasize that this value is based on our present experimental results, without further efforts to increase the MO contrast by optimizing the magnetic cladding composition and possible further improvements in the photonic design. Such engineering is expected to further boost the MO contrast and, consequently, theoretically achievable data rates.
